# Association of Maternal Metabolites and Metabolite Networks with Newborn Outcomes in a Multi-Ancestry Cohort

**DOI:** 10.3390/metabo13040505

**Published:** 2023-03-31

**Authors:** Brooke Gleason, Alan Kuang, James R. Bain, Michael J. Muehlbauer, Olga R. Ilkayeva, Denise M. Scholtens, William L. Lowe

**Affiliations:** 1 Feinberg School of Medicine, Northwestern University, Chicago, IL 60091, USA; 2 Duke Molecular Physiology Institute, Durham, NC 27701, USA; 3 Department of Medicine, Duke University School of Medicine, Durham, NC 27710, USA

**Keywords:** gestational diabetes, metabolomics, newborn adiposity

## Abstract

The in utero environment is important for newborn size at birth, which is associated with childhood adiposity. We examined associations between maternal metabolite levels and newborn birthweight, sum of skinfolds (SSF), and cord C-peptide in a multinational and multi-ancestry cohort of 2337 mother–newborn dyads. Targeted and untargeted metabolomic assays were performed on fasting and 1 h maternal serum samples collected during an oral glucose tolerance test performed at 24–32 week gestation in women participating in the Hyperglycemia and Adverse Pregnancy Outcome (HAPO) Study. Anthropometric measurements were obtained on newborns at birth. Following adjustment for maternal BMI and glucose, per-metabolite analyses demonstrated significant associations between maternal metabolite levels and birthweight, SSF, and cord C-peptide. In the fasting state, triglycerides were positively associated and several long-chain acylcarnitines were inversely associated with birthweight and SSF. At 1 h, additional metabolites including branched-chain amino acids, proline, and alanine were positively associated with newborn outcomes. Network analyses demonstrated distinct clusters of inter-connected metabolites significantly associated with newborn phenotypes. In conclusion, numerous maternal metabolites during pregnancy are significantly associated with newborn birthweight, SSF, and cord C-peptide independent of maternal BMI and glucose, suggesting that metabolites in addition to glucose contribute to newborn size at birth and adiposity.

## 1. Introduction

The prevalence of childhood obesity and associated metabolic disorders is increasing, indicating the need for early identification of those at risk to allow for early preventative interventions [1]. Childhood adiposity results from a combination of genetic and environmental factors, including the intrauterine environment [2,3]. Pregnancy is characterized by changes in carbohydrate, fat, and protein metabolism to match the physiological demands of pregnancy and ensure appropriate fetal development [4]. Pregnancy-related changes in metabolism, including those accompanying altered insulin sensitivity and fat accumulation, are reflected in the maternal metabolome [5]. The metabolome is influenced by both intrinsic (e.g., genetic) and extrinsic (e.g., diet, exercise, etc.) factors [6,7]. Understanding intrauterine metabolic predictors of newborn adiposity might help early identification of children at risk of developing childhood obesity and inform directed prevention efforts during pregnancy.

Newborn adiposity is a strong predictor of childhood adiposity. There is an independent positive association between newborn adiposity, as measured by sum of skin folds (SSF) and fat mass, and childhood adiposity [2,8]. Since newborn adiposity is a strong predictor for childhood adiposity, numerous studies have examined the relationship of the intrauterine environment to newborn adiposity [9,10]. High maternal glucose and BMI are two well-described factors independently associated with greater newborn adiposity [11]. Recent studies have shown that maternal hyperglycemia and obesity are also associated with childhood adiposity and metabolic health 10–14 years after delivery [8,12]. Metabolomic studies examining the relationship between maternal phenotype and maternal metabolites revealed that maternal BMI and hyperglycemia are associated with distinctive subsets of circulating metabolites [13]. Analyses of maternal circulating metabolites also indicated that metabolites beyond glucose contribute to newborn adiposity. Specifically, maternal levels of amino acids, acylcarnitines, lipids, and fatty acids and their metabolites during pregnancy are associated with fetal growth and adiposity, independent of maternal BMI and blood glucose levels [14]. As new metabolomic data have become available from the Hyperglycemia and Adverse Pregnancy Outcome (HAPO) Study, we re-examined the relationship between maternal metabolites and newborn birthweight, sum of skinfolds (SSF) and cord C-peptide to more fully characterize the maternal metabolome associated with newborn anthropometric outcomes.

## 2. Materials and Methods

### 2.1. Data and Sample Collection

The HAPO Study was a large, multinational, racially and ethnically diverse population-based study [15,16]. In brief, maternal blood samples were obtained during a 75 g oral glucose tolerance test (OGTT) at 24–32 weeks of gestation. Fasting, 1 h, and 2 h plasma glucose levels were measured. Caregivers and study participants were blinded to the OGTT results unless the fasting plasma glucose level was greater than 105 mg/dL or the 2 h plasma glucose level was greater than 200 mg/dL. Maternal serum samples were stored at −80 degrees C prior to metabolomics assays. Metabolic profiling was performed on maternal fasting and 1 h serum samples from 2337 mothers at 11 field centers worldwide. The field centers included Bangkok, Barbados, Belfast, Bellflower, Brisbane, Chicago, Cleveland, Hong Kong, Manchester, Newcastle, and Toronto.

The study protocol was approved by the Institutional Review Board at all participating sites, and each mother provided written informed consent. Trained personnel acquired maternal anthropometric measurements using a standardized protocol at the time of the OGTT. Gestational age was calculated from the date of the last menstrual period as described previously [15,16]. Demographic data were collected via questionnaire. Newborn anthropometric measurements, including birthweight and SSF, were measured within 72 h of delivery using calibrated equipment and standardized methods across field centers. Cord blood was obtained for measurement of serum C-peptide and other metabolites [15,16].

### 2.2. Conventional Metabolites and Targeted Metabolomics Assays

Conventional clinical metabolite and targeted metabolomics assays were conducted as previously described using maternal serum obtained fasting and 1 h after a glucose load [17,18]. Briefly, 15 amino acids, 45 acylcarnitines and 5 conventional clinical and targeted metabolites were analyzed. Levels of conventional clinical metabolites, including lactate, triglycerides, 3-hydroxybutyrate, glycerol, and nonesterified fatty acids (NEFA), were measured on a Unicel DxC 600 clinical analyzer (Beckman Coulter, Brea, CA, USA). Targeted metabolomics assays for acylcarnitines and amino acids were conducted by tandem mass spectrometry (MS) with addition of known quantities of stable isotope-labeled internal standards on an Acquity TQD Triple Quadrupole system (Waters Corporation, Milford, MA, USA).

### 2.3. Untargeted Metabolite Analyses

Untargeted metabolomics assays were performed using gas chromatography-mass spectrometry (GC-MS) to examine a broad range of maternal metabolites in serum. For these assays, maternal fasting and 1 h serum samples were extracted with methanol that was spiked with a retention time-locking (RTL) internal standard of perdeuterated myristic acid. Extracts were dried and derivatized by methoximation and trimethylsilylation [17,18]. Samples were run in daily batches of matched sets of fasting and 1 h maternal OGTT sera on a 7890B GC/5977B MS (Agilent Technologies, Santa Clara, CA, USA). To adjust for batch-to-batch and run order variation, quality control (QC) pools were established using equal volumes from all maternal samples and prepared for analysis as described above. QCs were injected as the first, middle and last samples of each daily GC-MS run. To control for technical variability attributable to batch and run order, GC-MS data were normalized using QC data and the Metabomxtr R package, version 1.16.0.6 [19]. GC-MS peaks were de-convoluted with Automated Mass Spectral Deconvolution and Identification System (AMDIS) freeware and annotated using the Agilent Fiehn GC-MS Metabolomics RTL spectral library, with additions from the laboratory at Duke University School of Medicine. Manual curation included identifying co-eluting groups of isomeric metabolites and selecting reliable peaks. Detected peak areas were log_2_-transformed for analysis. A total of 73 GC-MS metabolites not assayed using targeted approaches were included for data analysis.

### 2.4. Statistical Analyses

Acylcarnitine and 3-hydroxybutyrate levels were log-transformed to improve normality. Outlying metabolite values, defined as five or more SDs from the mean, and data from individuals with more than ten outlying metabolites for a sample type (fasting, 1 h) were excluded from analysis.

Associations between phenotypes and metabolites with <10% missing data within sample type were identified using linear regression within ancestry groups. Analyses treating maternal metabolites as predictors and newborn birthweight, SFF and cord C-peptide as outcomes were adjusted for field center, first 3 principal components of genetic ancestry [20], maternal mean arterial pressure, age, parity (0/1+), height, and smoker or drinker, newborn sex and gestational age at delivery, and sample storage time (Model 1). An additional model adjusted for all the covariates above plus maternal BMI and glucose during the HAPO OGTT (fasting for fasting metabolites and 1 h glucose for 1 h metabolites) (Model 2).

Graphical lasso techniques were used to identify the network structures underlying maternal metabolite associations with newborn phenotypes [21,22]. Metabolites were represented by nodes, and edges depicted non-zero partial correlations of metabolite pairs conditional on all other metabolites in the network. We identified a set of fasting maternal metabolites with less than 1% missing data and estimated the graph structure using the residuals from linear models with fasting metabolites as outcomes and Model 2 covariates as predictors, using graphical lasso through a sequence of values for a penalty term, *lambda*, where a stronger *lambda* results in a sparser network with fewer edges. The final *lambda* penalty term was selected using the rotation information criterion approach. Nodes on the resultant estimated network are sized according to the −log_10_(nominal *p*-value) from the fasting maternal per-metabolite analyses with newborn outcomes. To further elucidate network structure, spin-glass clustering was applied to the estimated networks to identify communities of nodes that are more tightly connected to each other than the other nodes in the network [23]. Similar approaches were applied to the maternal one-hour metabolite networks.

## 3. Results

### 3.1. Study Population

The characteristics of the 2337 participating mothers and their children appear in Table 1. The mean maternal BMI at OGTT was 27.9 and the mean fasting and 1 h glucose levels were 4.5 mmol/L and 7.5 mmol/L, respectively. Neonates were roughly equally distributed between males and females.

### 3.2. Associations of Maternal Metabolites with Newborn Phenotypes

The association of fasting and 1 h maternal metabolites with newborn birthweight, SSF, and cord C-peptide was examined. The β estimates and FDR-adjusted *p*-values for associations between maternal fasting and 1 h metabolites and newborn birthweight, sum of skinfolds, and cord C-peptide after adjustment for maternal BMI and glucose (Model 2) are shown in Table 2. Few fasting maternal metabolites were significantly associated with birthweight at baseline, but several were significantly associated in the fully adjusted model. Fasting alanine, pyruvic acid, and triglycerides were associated with birthweight in Model 1, as were two long-chain acylcarnitines, AC 14:2 and AC C18:2, and these associations persisted after adjustment for maternal BMI and fasting glucose (Model 2). Fasting 1,5-anhydroglucitol was negatively associated with birthweight in both the baseline and fully adjusted models. Several maternal metabolites became significantly associated with birthweight in the fully adjusted model. Specifically, arginine as well as acetylcarnitine and the medium-chain acylcarnitines AC C8:1, AC C8:1-OH/C6:1, AC 10:1, and AC C12:1 were negatively associated and methionine was positively associated with birthweight in the fully adjusted model (Model 2). Interestingly, these associations, which were not evident in the baseline model, became significant when maternal BMI was included in the model.

In contrast to the fasting state, several maternal metabolites 1 h after a glucose load were associated with birthweight in the baseline model (Model 1). These included the branched-chain amino acids (BCAA), although their association was attenuated after adjustment for maternal BMI and 1 h glucose (Model 2). Additional maternal amino acids, including alanine, threonine, methionine, proline, tyrosine, and glutamine/glutamic acid, were significantly associated with birthweight at baseline, with alanine, threonine, methionine, and proline remaining associated with birthweight in the fully adjusted model. Glycine was not significantly associated with birthweight at baseline, but it was significantly associated after adjustment for maternal BMI and 1 h glucose. Glucose and other aldohexoses, palmitoleic acid, pyruvic acid, 3-hydroxybuturate, and lactate were associated with birthweight at baseline, but these associations were attenuated after full adjustment. In contrast, triglycerides and 6-deoxyhexose were associated with birthweight both at baseline and after adjustment for maternal BMI and 1 h glucose. 1,5-anhydroglucitol was again negatively associated with birthweight in both the baseline and fully adjusted models, while a significant association of acetylcarnitine and the medium-chain acylcarnitine, AC C8:1, with birthweight became apparent after adjustment for maternal BMI and 1 h glucose.

Several fasting and 1 h maternal metabolites were associated with newborn SSF at baseline (Model 1), but many of these associations were again attenuated after adjustment for maternal BMI and fasting glucose (1 h glucose for 1 h metabolites). In the fasting state, the BCAAs, leucine/isoleucine and valine, as well as the BCAA metabolite, AC C5:1, were associated with SSF folds in Model 1. The association of fasting leucine/isoleucine and valine with SSF was attenuated after adjustment for maternal BMI and fasting glucose, although valine remained associated with SSF after adjustment for maternal BMI only. The association of AC C5:1 with SSF was present both in the baseline model and after adjustment for maternal BMI and fasting glucose (Model 2). Acetylcarnitine (AC C2), as well as several long-chain acylcarnitines, AC C14:2, AC C18:2, AC C16:2, and AC 16:1, were negatively associated with SSF after adjustment for maternal BMI, but of these, only AC C14:2 remained associated after adjustment for both maternal BMI and fasting glucose. Fasting 1.5-anhydroglucitol was negatively associated with SSF after adjustment for maternal BMI and fasting glucose, while fasting triglycerides were positively associated with SSF after adjustment for maternal BMI and fasting glucose.

One hour following a glucose load, the BCAAs and the BCAA metabolite, AC C5:1, together with triglycerides, were associated with SSF after adjustment for maternal BMI and 1 h glucose. Amino acids alanine, threonine, and proline were also associated with SSF after adjustment for maternal BMI and 1 h glucose, as were hydroxyprolines. 1,5-Anhydroglucitol was again negatively associated with SSF, in both the baseline and fully adjusted models. Pyruvic acid was associated with SSF in Model 1, but this association was attenuated after adjustment for maternal BMI and 1 h glucose. Several maternal acylcarnitines were not associated with SSF in the baseline model but were significantly and negatively associated with SSF after adjustment for maternal BMI and 1 h glucose. These included acetylcarnitine (AC C2), as well as the medium-chain acylcarnitine, AC C10-OH/C8-DC, and the long-chain acylcarnitines AC C14:2, AC C14:1, AC C14:1-OH, AC C20-OH/C18-DC, and AC C16:1.

A number of maternal fasting and 1 h metabolites were associated with cord C-peptide after adjustment for standard covariates (Model 1), but most of these associations were attenuated after further adjustment for maternal BMI and fasting glucose (1 h glucose for 1 h metabolites) (Model 2). In the fasting state, the branched-chain amino acids, leucine/isoleucine and valine, and their metabolites, AC C5 and AC C5-OH/C3-DC, together with triglycerides were associated with cord C-peptide in Model 1, but these associations were attenuated after adjustment for maternal BMI and glycemia. One hour after a glucose load, the BCAAs, together with their metabolite, ketoleucine/ketoisoleucine, and triglycerides were again associated with cord C-peptide; valine, ketoleucine/ketoisoleucine, and triglycerides remained associated with cord C-peptide after adjustment for maternal BMI and glycemia (Model 2).

### 3.3. Network Analyses

**Figure 1:**
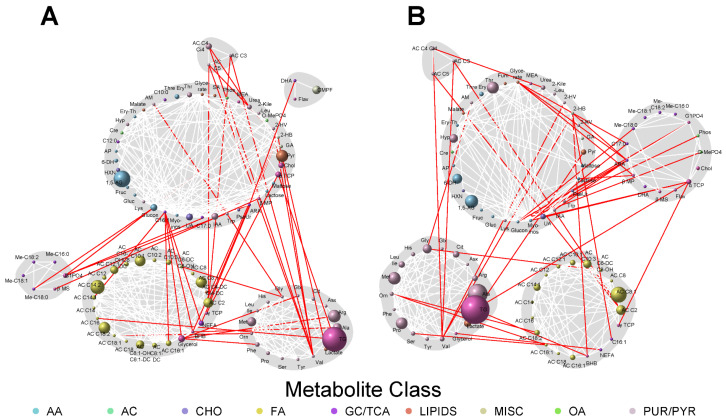
Maternal fasting (**A**) and 1 h (**B**) metabolite networks associated with birthweight. Grey shading represents spin-glass communities within the metabolite networks. The lines between two nodes (edges) represent dependence among metabolite pairs conditional on all other metabolites in the network according to graphical lasso. Red edges represent dependencies for metabolites in different spin-glass clusters and white edges represent dependencies for metabolites within the same spin-glass cluster. The node size of each individual metabolite reflects the strength of the association with each newborn outcome. A larger node size indicates a more robust association. Nodes are colored by metabolite class (AA, amino acid; AC, acylcarnitine; CHO, carbohydrate; FA, fatty acid; GC/TCA, glycolysis, tricarboxylic acid cycle; LIPIDS, lipids; MISC, miscellaneous; OA, organic acid; PUR/PYR, purine/pyrimidine). Metabolite names are abbreviated as follows: BHB, 3-Hydroxybuturate; TG, triglycerides; Pyr, pyruvic acid; GA, glycolic acid; 2-HB, 2-Hydroxybutyric acid; p-Cr, p-Cresol; 2-HV, 2-Hydroxyvaleric acid; O-MePO4, O-Methylphosphate; 2-Kile, 2-Ketoleucine; MEA, Ethanolamine; Phos, Phosphoric acid; SA, Succinic acid; C9:0, Nonanoic acid; Thre Ery, Threose erythrose; C10:0, Decanoic acid; AM, Aminomalonic acid; Hyp, Hydroxyprolines; Cre, Cretainine; C12:0, Lauric acid; AP, Aldopentoses; 6-DH, 6-Deoxyhexose; G1PO4, Glycerol 1-phosphate; HXN, Hypoxanthine; 1,5-AG, 1,5-Anhydroglucitol; Fruc, Fructose; Gluc, Glucose and other aldohexoses; Me-C16:0, Methyl palminate; Glucon, Gluconic acid; C16:1, Palmitoleic acid; Myo-inos, Myoinositol; UA, Uric Acid; Me-C18:2, Methyl linoleate; Me-C18:1, Methyl oleate; Me-C18:0, Methyl stearate; C17:0, Heptadecanoic acid; C18:0 Octadecanol; IAA, 3-Indolelactic acid; Trp, Tryptophan; PseUr, Pseudouridine; ARA, Arachidonic acid; β MP, beta-monoplamitin; Flav, Flavonoid short-chain cholesterol ester; γ TCP, gamma-Tocopherol; α TCP, alpha-Tocopherol; Chol, Cholesterol; 2-KV, 2-Ketovaline; Fum, Fumarate.

**Figure 2:**
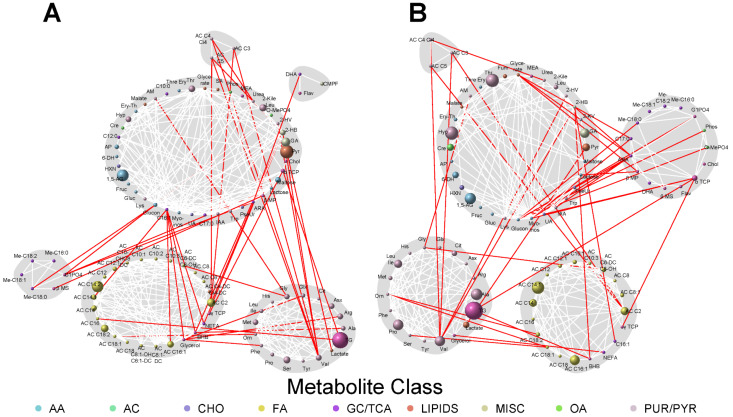
Maternal fasting (**A**) and 1 h (**B**) metabolite networks associated with newborn sum of skinfolds. Grey shading represents spin-glass communities within the metabolite networks. The lines between two nodes (edges) represent dependence among metabolite pairs conditional on all other metabolites in the network according to graphical lasso. Red edges represent dependencies for metabolites in different spin-glass clusters and white edges represent dependencies for metabolites within the same spin-glass cluster. The node size of each individual metabolite reflects the strength of the association with each newborn outcome. A larger node size indicates a more robust association. Nodes are colored by metabolite class as described in Figure 1. Metabolites are abbreviated as per the legend for Figure 1.

**Figure 3:**
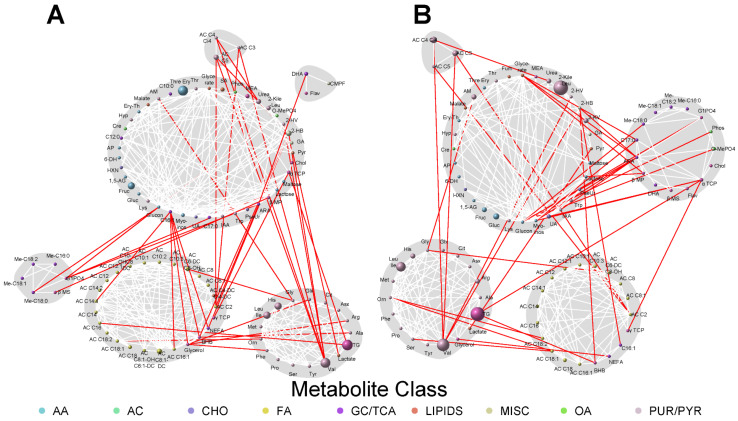
Maternal fasting (**A**) and 1 h (**B**) metabolite networks associated with cord C-peptide. Grey shading represents spin-glass communities within the metabolite networks. The lines between two nodes (edges) represent dependence among metabolite pairs conditional on all other metabolites in the network according to graphical lasso. Red edges represent dependencies for metabolites in different spin-glass clusters and white edges represent dependencies for metabolites within the same spin-glass cluster. The node size of each individual metabolite reflects the strength of the association with each newborn outcome. A larger node size indicates a more robust association. Nodes are colored by metabolite class as described in Figure 1. Metabolites are abbreviated as per the legend for Figure 1.

Because metabolites are interconnected, network analyses allow for the visualization of the greater context in which metabolite–phenotype associations occur. To that end, network analyses were conducted to identify joint associations of maternal metabolites at both fasting and 1 h with the newborn adiposity outcomes and cord C-peptide levels (Figure 1, Figure 2 and Figure 3). This enabled visualization of the connectivity between maternal metabolites that may or may not have demonstrated individually statistically significant associations with the newborn phenotypes. The size of each node reflected the strength of individual metabolite associations with each newborn outcome.

Several distinct metabolite communities were evident in the maternal metabolite networks associated with birthweight. At fasting, there were six distinct clusters of metabolites comprised of a variety of maternal metabolites, including fatty acids, carbohydrates, lipids, amino acids, and acylcarnitines. One large cluster included primarily medium- and long-chain acylcarnitines together with acetylcarnitine and additional lipids, including glycerol, non-esterified fatty acids, and 3-hydroxybutyrate. A second smaller cluster of acylcarnitines included the BCAA metabolites, AC C3, AC C4 and AC C5. Each acylcarnitine in this cluster had a connection to valine, which was included in a cluster of BCAAs and additional amino acids, most prominently alanine, methionine, and arginine. Other important metabolites in this cluster of amino acids were the triglycerides which, in turn, were connected to the large cluster of acylcarnitines through AC C16, palmitoylcarnitine. An additional large cluster contained metabolites from a variety of different classes with varying strengths of association (as reflected by different node sizes). Prominent members of this cluster included 1,5-anhydroglucitol and pyruvic acid.

At 1 h, there were five distinct clusters of maternal metabolites associated with birthweight. Similar to the fasting state, there was a cluster of largely long- and medium-chain acylcarnitines together with acetylcarnitine, a cluster of amino acids together with triglycerides, a small cluster of BCAA metabolites, and a large cluster of mixed metabolites. There was a new, larger community of metabolites that included cholesterol and *alpha*-tocopherol, as well as several fatty acids. Also of note is that the size of various nodes changed between the fasting and 1 h networks, reflecting changes in the strength of individual maternal metabolite associations with birthweight, but the inter-connectivity between the different communities of metabolites was maintained.

Given the relationship between birthweight and SSF, the maternal metabolite communities, both fasting and 1 h after a glucose load, present in the networks for these two newborn phenotypes were similar. Connectivity between the communities was also similar, although the strength of association (i.e., node size) of some metabolites differed between birthweight and SSF.

Cord C-peptide had similar maternal metabolite communities, although the prominence of different communities, based on the strength of association of metabolites within the communities, differed from the networks for birthweight and SSF. In the fasting state, triglycerides remained a highly associated metabolite. Within that community of metabolites, valine was more strongly associated with C-peptide than birthweight or SSF, while a number of other amino acids showed lesser degrees of association with C-peptide. Similarly, the community of acylcarnitines showed a weaker association with cord C-peptide than the other newborn phenotypes. For the communities present 1 h after a glucose load, the major differences were evidence for stronger association of various BCAA metabolites, i.e., the community of acylcarnitines that are BCAA metabolites as well as ketoleucine/ketoisoleucine, in the large community of mixed metabolites, and the weaker association of metabolites in the community of acetylcarnitine (AC C2) together with medium- and long-chain acylcarnitines. Connectivity between communities within the fasting and 1 h cord C-peptide networks was similar to that present in the networks for birthweight and SSF.

## 4. Discussion

This study examined associations of maternal metabolites during pregnancy with newborn birthweight, SSF, and cord C-peptide in the multi-ethnic HAPO cohort. Previously, the HAPO Study demonstrated significant associations of maternal fasting and 1 h metabolites during pregnancy with birthweight, SSF, and cord C-peptide [14]. In the present study, associations of the maternal metabolome with these newborn outcomes were examined in a larger cohort with greater ancestral diversity to identify novel associations. The present study demonstrated new associations of numerous metabolites with newborn birthweight, SSF and/or cord C-peptide independent of maternal glucose and BMI than previously identified, consistent with the concept that metabolites beyond glucose contribute to an intrauterine environment that affects newborn adiposity.

To date, most studies examining metabolite levels during pregnancy have focused on fasting metabolites or metabolites from random blood draws; few have evaluated maternal metabolites both fasting and following a standard glucose load. Thus, the present study was uniquely positioned to examine the association between maternal metabolite levels, both fasting and following a glucose load, with newborn measures of adiposity. Furthermore, most earlier studies included cohorts that were relatively small and from a homogenous ancestral background. The larger and multi-ancestry HAPO cohort provided greater power and the ability to detect associations that were common across ancestry groups and not limited to a single ancestry group. A Dutch cohort of 976 mother–newborn dyads examined the association of maternal nonfasting metabolites at approximately 12.8 weeks gestation with fetal growth from first trimester onward [24]. Few maternal metabolites associated with infant anthropometrics at birth were identified, suggesting that maternal metabolites present later in gestation have a larger impact on fetal adiposity and/or that a larger cohort was needed to detect significant associations. A study of 940 mother–offspring pairs of Asian ancestry that examined the association of fasting maternal metabolites at 28 weeks of gestation with newborn adiposity identified 28 metabolites associated with newborn SSF independent of maternal BMI [25]. Interestingly, the study reported inverse associations between maternal plasma levels of amino acid-related metabolites and newborn SSF, which is different from our finding of a positive association of maternal amino acids with newborn adiposity. This incongruity may be due to differences in cohort size or differences in metabolite associations and body composition amongst ancestry groups. Compared to the earlier studies, the HAPO cohort was larger, and the relationship between maternal metabolites and newborn adiposity both fasting and following a glucose load was examined, providing insight into two different physiologic states. A separate multi-national cohort of 3598 mother–newborn pairs assessed the relationship between maternal metabolite levels at approximately 13 weeks of gestation and fetal growth trajectories during pregnancy categorized into early accelerating growth, late accelerating growth, faltering growth, and median growth tracking [26]. Numerous metabolites had a positive or negative association with fetal growth trajectories, further suggesting that the maternal metabolome greatly impacts fetal growth, although maternal BMI and glycemia were not accounted for in those analyses.

The relationship between fasting maternal triglycerides and newborn size independent of maternal BMI and glucose has been previously described [27,28,29]. The present study confirmed our previous finding of an association of maternal fasting and 1 h triglycerides with birthweight and SSF [14], while also demonstrating an association of both fasting and 1 h triglycerides with cord C-peptide. In a Chinese cohort of 5695 pregnant women, second and third trimester triglyceride levels were predictors of large-for-gestational-age infants [30]. A separate study assessed the correlation between fasting and postprandial maternal triglycerides at 14–16 weeks and 26–28 weeks of gestation with newborn percent body fat. In obese mothers, postprandial triglycerides demonstrated the strongest association with newborn percent fat, while in normal-weight mothers, the increase in triglycerides from early to mid-pregnancy demonstrated the strongest association with percent body fat at birth [31]. Our findings together with the results of these earlier studies suggest that maternal fuels beyond glucose contribute to excess fetal adiposity.

Consistent with our previous finding of an association of maternal BCAAs with newborn phenotypes, we demonstrated association of 1 h maternal leucine/isoleucine with SSF and 1 h maternal valine with cord C-peptide and SSF following adjustment for maternal BMI and glucose. Few studies beyond ours have examined the relationship between maternal BCAA levels and fetal growth, but the relationship between nutritional BCAA content and postnatal growth has been examined. Prior studies have suggested that circulating BCAAs promote growth by stimulating insulin and insulin-like growth factor (IGF-1) secretion [32,33]. Insulin and IGF-1 can modulate growth through activation of the mammalian target of rapamycin (mTOR) pathway. BCAAs can also activate mTOR through a pathway independent of insulin [34]. It is hypothesized that regulation of the mTOR pathway controls metabolism, bodyweight, and body composition [35,36]. Our finding that maternal BCAA levels are associated with SSF independent of maternal BMI and glucose is consistent with a model in which BCAAs crossing the placenta promote fetal growth through activation of the mTOR pathway. This model is also supported by our previous study demonstrating a positive association between leucine/isoleucine levels in cord blood and birthweight independent of glucose and BMI [14].

Higher proline levels have been detected in individuals with insulin resistance, obesity, and type 2 diabetes [37]. In the present study, after adjustment for BMI and glucose, 1 h maternal proline was associated with SSF and birthweight, while 1 h maternal hydroxyproline, a metabolite of proline, was associated with SSF in the fully adjusted model. Greater proline availability in the maternal plasma of pigs and sheep increases concentrations of proline and polyamines in the placenta and fetal fluids and promotes fetal growth [38]. Proline is a major building block for polyamines, which play a key role in cell proliferation, growth, and differentiation. Previous studies have demonstrated a significant effect of polyamines on fetal development [39]. Together with our findings, these studies suggest that higher levels of maternal proline promote fetal growth and contribute to fetal adiposity.

Acylcarnitine levels change throughout pregnancy reflecting altering metabolic requirements [40]. One study that examined associations of 28 acylcarnitines across gestation with newborn adiposity measures including birthweight and SSF determined that levels of C12, C14, C14:1 at gestational weeks 15–26, C6 at gestational weeks 23–31, and C4 and C16 at gestational weeks 33–39 were inversely associated with birthweight [41]. These findings are consistent with the present study, in which multiple maternal long-chain acylcarnitines were inversely associated with birthweight and SSF, both fasting and 1 h after a glucose load, following adjustment for BMI and maternal glucose. The greater number of associations between maternal acylcarnitines and newborn adiposity identified in the HAPO Study likely reflected increased power secondary to the larger cohort.

We noted the inverse associations between 1,5-anhydroglucitol (1,5-AG) and both birthweight and SSF. Depletion of circulating 1,5-AG, a sugar alcohol found in many foods of botanical origin, marks recent hyperglycemic events severe enough to have triggered glycosuria [42]. Previously, it was noted that 1,5-AG levels are inversely associated with 1 h but not fasting glucose levels during an OGTT to diagnose GDM [43]. These observations are consistent with our finding that maternal 1,5-AG was inversely associated with birthweight and newborn SSF.

Finally, consistent with the findings reported in this study, we previously demonstrated that maternal metabolites beyond glucose contribute to newborn birthweight and SSF [17]. Previous studies have demonstrated that pharmacologic treatment decreases the frequency of but does not completely prevent the short-term adverse maternal and newborn outcomes associated with GDM [44,45,46,47,48]. The impact of treatment of GDM on the maternal metabolome and adverse pregnancy outcomes has not been specifically examined, but previous studies have suggested that differences in the maternal metabolome persist despite pharmacologic treatment of GDM. White et al. examined the metabolome in obese women with GDM treated with insulin or metformin [49]. After ~7 weeks of treatment, both metformin and insulin improved the metabolic profile but differences in lipids and amino acids persisted compared to women without GDM. Similarly, Mokkala et al. examined overweight or obese women and compared the metabolome of women treated with insulin or metformin vs. diet only [50]. Treatment with metformin or insulin resolved differences in glucose between the pharmacologic treatment vs. diet treatment only groups; however, differences in 75 lipid variables, including triglycerides, total lipids and size of VLDL particles, as well as leucine and isoleucine were still present in women treated with metformin or insulin compared to diet alone. Our earlier and current results together with the previous studies described above may explain, in part, the inability of treatment to fully prevent the adverse outcomes associated with GDM.

The large, multi-ancestry HAPO cohort is a strength of this study, as it enabled identification of metabolite associations that are shared across ancestries. Use of SSF as a measure of newborn adiposity is also a strength, since SSF more accurately reflects adiposity than birthweight or ponderal index [51,52]. The availability of metabolite levels, both fasting and 1 h after a glucose load, allowed for examination of associations in two different metabolic states. A limitation of the study is an inability to monitor maternal metabolite levels at different time points during pregnancy, since our metabolite levels were taken at one time point, at approximately 28 weeks of gestation. Studies have suggested that fluctuations in metabolite levels during gestation may modulate fetal growth [53]. Furthermore, metabolites are impacted by multiple environmental factors (e.g., diet), many of which likely differed across field centers and could not be accounted for in the present study. However, the present study demonstrated that multiple maternal metabolites are significantly associated with newborn phenotypes across different environments and ancestry groups due to common physiologic, genetic and/or environmental factors.

Maternal metabolites during pregnancy, both fasting and 1 h post glucose load, were significantly associated with newborn adiposity and cord C-peptide after adjusting for maternal BMI and glucose. Maternal levels of triglycerides, amino acids, and acylcarnitines and their metabolites may impact fetal growth, adiposity, and insulin resistance. Further evaluation is needed to determine the mechanisms by which metabolites affect fetal growth as well as the impact of metabolite changes throughout gestation on adiposity.

## Figures and Tables

**Table 1:** Characteristics of participating mothers and newborns.

Maternal Participants (n = 2337)	
Black	663 (28.4%)
East Asian	436 (18.7%)
Hispanic	53 (2.3%)
South Asian	629 (26.9%)
White	556 (23.8%)
**Maternal Characteristics**	
Age at OGTT, years	29.2 (5.8)
Height, cm	161.0 (7.1)
BMI at OGTT, kg/m^2^	27.9 (5.1)
Mean arterial pressure, mmHg	80.7 (7.8)
Fasting plasma glucose, mmol/L	4.5 (0.4)
1 h plasma glucose, mmol/L	7.5 (1.7)
Gestational age at OGTT, weeks	27.8 (1.8)
Gestational age at delivery, weeks	39.8 (1.2)
Smoking (continued smoking in pregnancy)	61 (2.6%)
Alcohol (continued alcohol consumption in pregnancy)	103 (4.4%)
Parity (nulliparous)	1245 (53.3%)
**Newborn Participants (n = 2337)**	
Black	559 (23.9%)
East Asian	654 (28.0%)
Hispanic	451 (19.3%)
South Asian	50 (2.1%)
White	623 (26.7%)
**Newborn Characteristics**	
Birthweight, g	3391.5 (485.0)
SSF	12.5 (2.7)
Cord blood C-peptide, nmol/L	0.3 (0.2)
Male sex	1175 (50.3%)
Female sex	1162 (49.7%)

Data are shown as mean (SD) or n (%).

**Table 2:** Associations of maternal metabolites with newborn outcomes.

	Model 1 Beta (CI, *p*)	Model 2 Beta (CI, *p*)
	**Birthweight**	
	*Fasting metabolites*	
Arginine	−12.92 (−31.86–6.03, 0.35)	**−33.07 (−51.80–14.35, 1.18 × 10^−2^)**
Triglycerides	52.13 (34.53–69.73, 4.81 × 10^−7^) *	**44.80 (27.58–62.03, 2.43 × 10^−5^)**
Alanine	**30.31 (12.91–47.70, 1.86 × 10^−2^)**	**25.26 (7.97–42.55, 3.06 × 10^−2^)**
Methionine	22.07 (4.96–39.18, 8.13 × 10^−2^)	**24.51 (7.86–41.17, 3.06 × 10^−2^)**
Pyruvate	**32.57 (17.77–47.37, 8.90 × 10^−4^)**	**25.16 (10.64–39.67, 1.84 × 10^−2^)**
AC C2	−20.61 (−38.00–−3.22, 0.11)	**−26.21 (−43.22–−9.20, 2.37 × 10^−2^)**
AC C8:1	−10.74 (−28.18–6.69, 0.39)	**−28.54 (−45.81–−11.27, 1.58 × 10^−2^)**
AC C8:1-OH/C6:1-DC	−21.06 (−38.84–−3.29, 0.11)	**−24.14 (−41.45–−6.82, 4.12 × 10^−2^)**
AC C10:1	−22.05 (−39.08–−5.02, 8.13 × 10^−2^)	**−26.973 (−43.58–−10.36, 1.60 × 10^−2^)**
AC C12:1	−18.87 (−36.12–−1.63, 0.12)	**−23.01 (−39.93–−6.10, 4.57 × 10^−2^)**
AC C14:2	**−29.05 (−46.12–−11.99, 1.86 × 10^−2^**	**−31.70 (−48.42–−14.98, 6.76 × 10^−3^)**
AC C18:2	**−28.45 (−46.14–−10.76, 2.67 × 10^−2^)**	**−29.35 (−46.65–−12.06, 1.45 × 10^−2^)**
1,5-Anhydroglucitol	**−33.31 (−49.46–−17.17, 1.43 × 10^−3^)**	**−33.06 (−48.88–−17.23, 2.32 × 10^−3^)**
	*One-hour metabolites*	
Leucine/Isoleucine	**38.92 (21.57–56.26, 1.88 × 10^−4^)**	20.33 (3.10–37.55, 8.13 × 10^−2^)
Valine	**34.77 (17.04–52.50, 1.16 × 10^−3^)**	17.36 (−0.178–34.90, 0.13)
Triglycerides	**59.48 (41.75–77.20, 3.93 × 10^−9^)**	**49.66 (32.270–67.06, 1.61 × 10^−6^)**
Alanine	**42.74 (25.18–60.30, 6.35 × 10^−5^)**	**39.81 (22.68–56.94, 1.79 × 10^−4^)**
Glutamine/Glutamic acid	**33.36 (15.40–51.33, 2.27 × 10^−3^)**	11.24 (−6.69–29.17, 0.36)
Methionine	**35.44 (18.39–52.48, 5.20 × 10^−4^)**	**29.51 (12.90–46.11, 8.21 × 10^−3^)**
Proline	**36.23 (19.17–53.30, 4.28 × 10^−4^)**	**28.53 (11.89–45.18, 1.03 × 10^−2^)**
Hydroxyprolines	**21.47 (9.88–33.07, 5.23 × 10^−3^)**	16.22 (4.91–27.53, 5.40 × 10^−2^)
Threonine	**23.25 (11.70–34.80, 2.64 × 10^−3^)**	**19.44 (8.19–30.68, 1.93 × 10^−2^)**
Tyrosine	**30.36 (13.20–47.51, 3.86 × 10^−3^)**	14.23 (−2.73–31.18, 0.20)
Glycine	11.89 (−5.77–29.56, 0.46)	**25.29 (8.02–42.56, 3.85 × 10^−2^)**
Pyruvate	**22.56 (9.52–35.60, 8.06 × 10^−3^)**	13.52 (0.73–26.31, 0.30)
AC C2	−4.05 (−21.81–13.71, 0.85)	**−27.04 (−44.66–9.43, 2.87 × 10^−2^)**
AC C6	**29.21 (10.17–48.25, 1.59 × 10^−2^)**	11.72 (−7.01–30.46, 0.36)
AC C8:1	−11.02 (−28.43–6.39, 0.50)	**−35.39 (−52.68–−18.09, 1.36 × 10^−3^)**
Lactate	**30.03 (11.80–48.26, 8.22 × 10^−3^)**	23.08 (5.11–41.06, 7.04 × 10^−2^)
3-Hydroxybutyrate	**39.80 (22.19–57.40, 1.88 × 10^−4^)**	3.83 (−14.60–22.25, 0.71)
Glucose and other aldohexoses	**27.01 (11.33–42.68, 8.06 × 10^−3^)**	−0.56 (−16.90–15.79, 0.95)
6-Deoxyhexose	**17.07 (5.98–28.17, 2.33 × 10^−2^)**	**16.67 (5.89–27.45, 4.46 × 10^−2^)**
3-Carboxy-4-methyl-5-propyl-2-furanpropanoic acid	**−16.99 (−28.95–−5.03, 4.04 × 10^−2^)**	−16.65 (−28.26–−5.04, 5.40 × 10^−2^)
1,5-Anhydroglucitol	**−31.51 (−47.33–−15.69, 2.64 × 10^−3^)**	**−28.51 (−44.07–−12.95, 1.82 × 10^−2^)**
Palmitoleic acid	**11.63 (3.35–19.92, 4.04 × 10^−2^)**	3.96 (−4.23–12.15, 0.71)
	**Sum of Skinfolds**	
	*Fasting metabolites*	
Leucine/Isoleucine	**0.15 (0.05–0.26, 4.09 × 10^−2^)**	0.09 (−0.01–0.19, 0.25)
Valine	**0.21 (0.10–0.32, 4.26 × 10^−3^)**	0.12 (0.01–0.22, 0.16)
Triglycerides	**0.25 (0.15–0.36, 1.70 × 10^−4^)**	**0.21 (0.11–0.31, 4.84 × 10^−3^)**
Alanine	**0.15 (0.05–0.25, 4.09 × 10^−2^)**	0.10 (−0.01–0.20, 0.23)
Proline	**0.16 (0.06–0.26, 3.57 × 10^−2^)**	0.09 (−0.02–0.19, 0.28)
Glycine	**−0.15 (−0.26–−0.05, 4.09 × 10^−2^)**	−0.11 (−0.21–−0.00, 0.17)
Pyruvate	**0.20 (0.11–0.29, 5.39 × 10^−4^)**	**0.16 (0.07–0.25, 2.05 × 10^−2^)**
AC C5:1	**0.18 (0.07–0.28, 1.68 × 10^−2^)**	**0.17 (0.07–0.27, 2.33 × 10^−2^)**
AC C14:2	**−0.16 (−0.26–−0.06, 3.45 × 10^−2^)**	**−0.17 (−0.26–−0.07, 2.74 × 10^−2^)**
1,5-Anhydroglucitol	**−0.17 (−0.26–−0.07, 1.72 × 10^−2^)**	**−0.16 (−0.25–−0.06, 2.70 × 10^−2^)**
	*One-hour metabolites*	
Leucine/Isoleucine	**0.23 (0.12–0.34, 2.72 × 10^−4^)**	**0.14 (0.04–0.24, 4.73 × 10^−2^)**
Valine	**0.25 (0.14–0.36, 1.41 × 10^−4^)**	**0.17 (0.06–0.27, 1.85 × 10^−2^)**
Triglycerides	**0.29 (0.18–0.39, 1.32 × 10^−5^)**	**0.23 (0.12–0.33, 1.68 × 10^−3^)**
Alanine	**0.19 (0.09–0.30, 5.10 × 10^−3^)**	**0.17 (0.07–0.28, 1.85 × 10^−2^)**
Proline	**0.21 (0.10–0.31, 1.81 × 10^−3^)**	**0.16 (0.06–0.26, 1.85 × 10^−2^)**
Hydroxyprolines	**0.15 (0.08–0.21, 1.92 × 10^−3^)**	**0.12 (0.05–0.19, 1.21 × 10^−2^)**
Creatinine	**0.10 (0.03–0.17, 3.57 × 10^−2^)**	0.08 (0.02–0.15, 0.15)
Threonine	**0.14 (0.07–0.21, 2.28 × 10^−3^)**	**0.12 (0.05–0.19, 1.21 × 10^−2^)**
Glycolic acid	**−0.07 (−0.11–−0.02, 4.87 × 10^−2^)**	−0.07 (−0.11–−0.02, 5.03 × 10^−2^)
Pyruvate	**0.15 (0.07–0.23, 5.68 × 10^−3^)**	0.10 (0.02–0.18, 0.12)
AC C5:1	**0.16 (0.06–0.27, 2.13 × 10^−2^)**	**0.16 (0.06–0.26, 1.85e × 10^−2^)**
AC C2	−0.02 (−0.13–0.08, 0.80)	**−0.14 (−0.25–−0.04, 4.73 × 10^−2^)**
AC C10-OH/C8-DC	−0.07 (−0.17–0.04, 0.55)	**−0.16 (−0.27–−0.06, 1.85 × 10^−2^)**
AC C14:1	−0.04 (−0.14–0.07, 0.77)	**−0.18 (−0.28–−0.08, 1.85 × 10^−2^)**
AC C14:1-OH	−0.10 (−0.21–−0.00, 0.21)	**−0.15 (−0.25–−0.05, 2.55 × 10^−2^)**
AC C14:2	−0.04 (−0.15–0.06, 0.77)	**−0.17 (−0.27–−0.06, 1.85 × 10^−2^)**
AC C16:1	−0.03 (−0.14–0.07, 0.77)	**−0.16 (−0.26–−0.05, 2.11 × 10^−2^)**
AC C20-OH/C18-DC	−0.12 (−0.22–0.01, 1.69 × 10^−1^)	**−0.14 (−0.24–−0.03, 4.86 × 10^−2^)**
Lactate	**0.17 (0.06–0.28, 2.13 × 10^−2^)**	0.13 (0.02–0.23, 9.99 × 10^−2^)
3-Hydroxybutyrate	**0.17 (0.06–0.27, 2.13 × 10^−2^)**	−0.03 (−0.14–0.09, 0.72)
Glucose and other aldohexoses	**0.15 (0.05–0.24, 2.54 × 10^−2^)**	0.00 (−0.01–0.10, 0.99)
1,5-Anhydroglucitol	**−0.17 (−0.27–−0.08, 5.68 × 10^−3^)**	**−0.15 (−0.25–−0.06, 2.55 × 10^−2^)**
	**Cord C-Peptide**	
	*Fasting metabolites*	
Arginine	**0.04 (0.02–0.07, 2.67 × 10^−2^)**	0.019 (−0.01–0.04, 0.64)
Leucine/Isoleucine	**0.04 (0.02–0.07, 6.96 × 10^−3^)**	0.029 (0.01–0.05, 0.14)
Valine	**0.06 (0.03–0.08, 1.67 × 10^−4^)**	0.04 (0.01–0.06, 0.11)
Triglycerides	**0.05 (0.02–0.07, 4.28 × 10^−3^)**	0.04 (0.01–0.06, 0.11)
AC C5	**0.04 (0.01–0.06, 2.67 × 10^−2^)**	0.02 (0.00–0.05, 0.33)
AC C5-OH/C3-DC	**0.04 (0.02–0.07, 7.94 × 10^−3^)**	0.03 (0.01–0.06, 0.11)
	*One-hour metabolites*	
Arginine	**0.04 (0.01–0.06, 4.26 × 10^−2^)**	0.02 (−0.00–0.05, 0.47)
Leucine/Isoleucine	**0.05 (0.03–0.08, 3.49 × 10^−4^)**	0.04 (0.01–0.06, 6.34 × 10^−2^)
Valine	**0.06 (0.03–0.08, 1.92 × 10^−4^)**	**0.04 (0.02–0.07, 2.31 × 10^−2^)**
Triglycerides	**0.06 (0.03–0.08, 1.92 × 10^−4^)**	**0.05 (0.02–0.07, 1.86 × 10^−2^)**
3-Hydroxybutyrate	**0.04 (0.02–0.07, 1.01 × 10^−2^)**	0.01 (−0.02–0.03, 0.85)
Glucose and other aldohexoses	**0.05 (0.03–0.07, 1.41 × 10^−4^)**	0.03 (0.00–0.05, 0.56)
2-Ketoleucine/ketoisoleucine	**0.03 (0.02–0.04, 8.03 × 10^−4^)**	**0.03 (0.01–0.04, 1.17 × 10^−2^)**

* Betas, confidence intervals and *p* values in bold font reflect significant associations of maternal metabolites with newborn birthweight, sum of skinfolds, and cord C-peptide after correction for false discovery.

## Data Availability

The data presented in this study are available on request from the corresponding author. Metabolomic data and codes used for analyses will be made available by the authors on request. Data will soon be publicly available through the Northwestern University DigitalHub.
